# Transanal total mesorectal excision (TaTME) for rectal cancer: effects on patient-reported quality of life and functional outcome

**DOI:** 10.1007/s10151-016-1570-z

**Published:** 2017-01-02

**Authors:** T. W. A. Koedam, G. H. van Ramshorst, C. L. Deijen, A. K. E. Elfrink, W. J. H. J. Meijerink, H. J. Bonjer, C. Sietses, J. B. Tuynman

**Affiliations:** 1 Department of Surgery, VU University Medical Center, De Boelelaan 1117, 1081 HV Amsterdam, The Netherlands; 2 Department of Surgery, Antoni van Leeuwenhoek Hospital Netherlands Cancer Institute, Amsterdam, The Netherlands; 3 Department of Surgery, Hospital Gelderse Vallei, Ede, The Netherlands

**Keywords:** Rectum, Cancer, Surgery, TaTME, Quality of life, PROMs

## Abstract

**Background:**

Transanal total mesorectal excision (TaTME) has rapidly become an important component of the treatment of rectal cancer surgery. Cohort studies have shown feasibility concerning procedure, specimen quality and morbidity. However, concerns exist about quality of life and ano(neo)rectal function. The aim of this study was to prospectively evaluate quality of life in patients following TaTME for rectal cancer with anastomosis.

**Methods:**

Consecutive patients who underwent restorative TaTME surgery for rectal adenocarcinoma in an academic teaching center with tertiary referral function were evaluated. Validated questionnaires were prospectively collected. Quality of life was assessed by the EuroQol 5D (EQ-5D), European Organization for Research and Treatment of Cancer’s QLQ-C30 and QLQ-CR29 and low anterior resection syndrome (LARS) scale. Outcomes of the questionnaires at 1 and 6 months were compared with preoperative (baseline) values.

**Results:**

Thirty patients after restorative TaTME for rectal cancer were included. Deterioration for all domains was mainly observed at 1 month after surgery compared to baseline, but most outcomes had returned to baseline at 6 months. Social function and anal pain remained significantly worse at 6 months. Major LARS (score >30) was 33% at 6 months after ileostomy closure. No end colostomies were required.

**Conclusions:**

TaTME is associated with acceptable quality of life and functional outcome at 6 months after surgery comparable to published results after conventional laparoscopic low anterior resection.

## Introduction

The transanal total mesorectal excision (TaTME) technique for mid- and low rectal cancer has raised great interest worldwide. Available data from cohort studies suggest that TaTME is a feasible and safe technique [1–4]. The overall morbidity, specimen quality and margins appear to be comparable to conventional low anterior resection, while high-quality data from randomized studies are still lacking [5–7]. Moreover, long-term outcome is still unknown.

The transanal approach enhances visualization of the surgical planes in the mid- and low mesorectum, allowing more careful dissection compared to conventional TME. Potential advantages of the TaTME technique are less morbidity, less conversions to open or Hartmann’s procedure and more sphincter saving procedures. However, concerns exist whether the TaTME technique hampers functional outcome compared to conventional low anterior resection. First, the level of anastomosis in TaTME is potentially created closer to the anal sphincter compared to conventional laparoscopic TME. Secondly, the TaTME technique could result in damage to the sphincter caused by prolonged dilatation of the anal canal. At last, in TaTME, the resection could be more radical in the lower pelvis, especially within the learning phase, which could be associated with collateral damage to the innervation of the levator ani muscle. Currently, data on quality of life and ano(neo)rectal function after TaTME are scarce [8–12]. The published studies have used a variety of scores and methods to define and compare fecal incontinence, urinary and sexual dysfunction. These studies also lack comparison with preoperative baseline values.

To our knowledge, this study is the first to present prospectively reported quality of life of patients undergoing TaTME low anterior resection for rectal cancer using validated questionnaires.

## Materials and methods

VU University Medical Center in Amsterdam is an academic teaching hospital with a tertiary referral function for patients with rectal cancer. Patients presented with rectal cancer in the VU University Medical Center between January 2014 and January 2016 and operated on using TaTME with construction of a primary anastomosis after resection were included. Preoperative work-up including imaging and neoadjuvant treatment was organized according to the Dutch National Guidelines for Rectal Cancer, and postoperative care according to Enhanced Recovery after Surgery (ERAS) guidelines. The technique used has been described in an earlier article. An ileostomy was created when deemed necessary by the treating surgeon, and after 6 weeks the anastomosis was evaluated by a computed tomography (CT) scan with contrast enema and under direct visualization using sigmoidoscopy. After excluding anastomotic problems, the stoma was reversed. Only patients with good preoperative sphincter function were selected for low anastomosis, assessed by history taking, digital examination and additional manometry when the treating surgeon judged objective confirmation of the function was needed. All patients undergoing abdominoperineal resection (APR), operations for benign disease or follow-up less than 6 months were excluded. This study was approved by the Institutional Review Board of VU University Medical Center.

### Baseline data

Baseline characteristics such as gender, age, body mass index (BMI), neoadjuvant treatment, clinical American Joint Committee on Cancer (AJCC) stage were prospectively collected from patient records. Operative and postoperative data included type of anastomosis, creation of diverting ileostomy, type of specimen extraction, length of hospital stay, and complications during and after surgery including bleeding, technical problems, urinary retention, anastomotic leakage (definition according to Rahbari et al. [13]) diagnosed on CT scan, coloanal stenosis needing dilatation, and stoma-related problems. Regarding pathology outcomes, AJCC stage, quality of specimen (using Quirke’s classification [14]), circumferential mesorectal margin (CRM) involvement, total number of lymph nodes harvested, tumor diameter, and radicality were collected. During follow-up, the following data were collected: time to local recurrence defined as tumor found near the anastomosis, time to distant recurrence defined as metastasis in other organ(s) and survival. Follow-up was organized according to the Dutch National Guidelines for Colorectal Cancer.

### Questionnaires

Patient-reported outcome measurements were collected prospectively by sending questionnaires within 1 week before surgery and 1 and 6 months after surgery. The following questionnaires were used: EuroQol with five dimensions (EQ-5D-3L), European Organization for Research and Treatment of Cancer (EORTC) QLQ-CR29 and QLQ-C30, version 3.0, and low anterior resection syndrome (LARS).

The EQ-5D-3L questionnaire was used to evaluate the level of mobility, self-care, activity, pain, and anxiety. The EQ-5D index was calculated for overall scoring and the EQ-VAS [a visual analogue scale from 0 (worst) to 100 (best)] was used to asses patients’ global health.

The *EORCT QLQ*-*CR29 module* was analyzed using 4 multi-item scales including body image (items 45, 46 and 47), micturition scale (items 31 and 32), blood and mucus in stool (items 38 and 39), frequency of bowel movement (items 52 and 53) and 19 single items according to modifications by Whistance et al. [15].

For the *EORCT QLQ*-*C30* module scoring procedures were used as described in the EORTC scoring manual QLQ-C30, version 3.0 [16]. The QLQ-C30 is composed of 5 functional scales, 3 symptom scales, a global health status and 6 single items.

The QLQ-CR29 and QLQ-C30 were converted to a score ranging from 0 to 100 in order to compare means. Changes in QLQ-C29 and CR30 were interpreted to be small, moderate and substantial if difference in mean scores was 5–10, 10–20 and greater than 20, respectively [17].

**Fig. 1:**
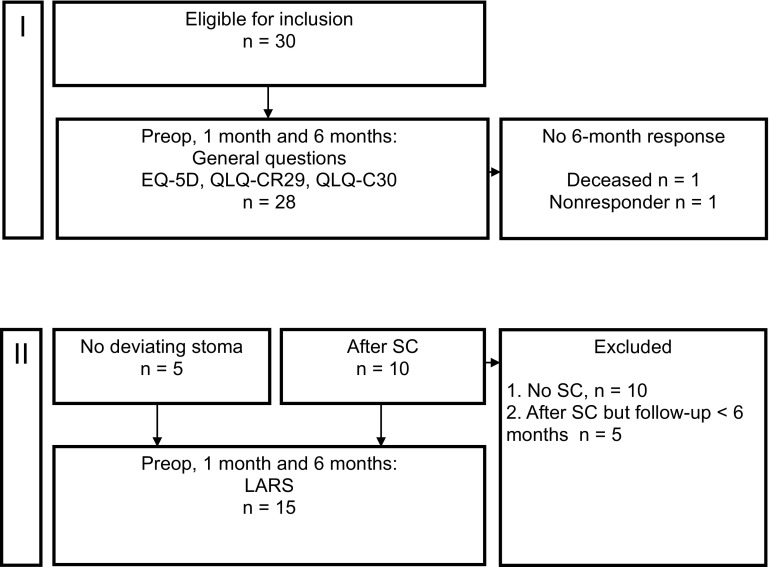
Overview included patients in the general analysis (*I*) and ano(neo)rectal function (*II*). *SC* stoma closure, *LARS* low anterior resection syndrome

To evaluate the ano(neo)rectal function after TaTME, the LARS questionnaire was used. In case of an ileostomy, the questionnaire was sent 1 and 6 months after stoma closure (Fig. 1). The LARS score was categorized into no LARS (0–20 points), minor LARS (21–29 points), and major LARS (30–42 points) [18].

### Statistical analysis

Repeated measures analysis of variance (ANOVA) was used to investigate changes in mean questionnaire scores between 3 time points. In case Mauchly’s Test of sphericity was violated, we used the Greenhouse-Geisser correction when the estimate of sphericity (*ε*) was <0.75 and the Huynh–Feldt correction when ε was >0.75. If the effect was significant, post hoc testing using the Bonferroni correction was performed in order to specify the statistical significant difference between the measurement moments. A *p* value ≤0.05 was considered statistically significant. Statistical analysis was done using SPSS version 23 for Windows and Mac (SPSS, Chicago, Illinois, USA).

## Results

Between January 2014 and February 2016, 140 patients underwent rectal resection at VU University Medical Center. Seventy-one of these patients underwent elective resection with curative intent for rectal cancer. The other patients underwent resection for benign or metastasized disease. Thirty consecutive patients undergoing TaTME with a primary anastomosis were included for prospective collection of questionnaires. The 30 patients (21 males, 70%) had a median age of 65 years (interquartile range (IQR) 58–69 years), the mean BMI was 26 kg/m^2^ (IQR 24–28 kg/m^2^) and 27 (90%) patients were categorized as American Society of Anesthesiologists (ASA) class I–II, (Table 1). Median tumor distance from the anal verge on magnetic resonance imaging (MRI) was 6 cm (IQR 4–8 cm). Twenty-two patients (73%) received neoadjuvant treatment; 12 patients (40%) short course radiotherapy and 10 patients (33%) long course chemoradiotherapy. One patient had salvage TaTME for a local regrowth during wait and see policy after initial complete response on neoadjuvant therapy. Three patients underwent completion TaTME after local excision of the primary tumor.

**Table 1:** Patient and tumor characteristics

Age (median, SD, years)	66 (9.3)
Male	21 (70%)
Body mass index (median, SD, kg/m2)	26.4 (3.8)
Tumor height from anal verge (median, IQR, cm)	6.0 (4.0–8.0)
AJCC stage	
0	4 (13)
I	11 (37)
II	6 (20)
III	7 (23)
IV	2 (7)
Neoadjuvant therapy	22 (73)
Radiotherapy only	12 (40)
Chemoradiotherapy	10 (33)
No diverting ileostomy at index surgery	6 (20)

Male, neoadjuvant therapy, AJCC stage and ileostomy are presented as frequency with percentage

*SD* standard deviation, *IQR* interquartile range, *AJCC* American Joint Committee on Cancer

At the time of analysis, 15 of the 25 diverting ileostomies were reversed, and the median period after primary surgery was 4 months (IQR 3–5 months). Median follow-up after reversal was 11 months (IQR 5–16 months). Stoma was not reversed due to a chronic presacral sinus in the 3 patients, metastatic disease in 1 patient needing adjuvant chemotherapy, preference to wait before undergoing another operation after an earlier correction of the stoma in one patient, and death at 7 months after removal of the rectum in 1 patient. At the time of analysis, reversal had been planned for 4 patients.

### Response rate

The response rate at 6 months was 93% for the general population (*n* = 28) and 100% for the population analyzed after stoma closure (*n* = 15). One patient died at 7 months and another patient was unwilling to fill out the last questionnaire, therefore, no 6-month questionnaires were received from these patients.

### Operative details

All included patients received primary anastomosis, performed by circular stapler in 17 patients (57%) and hand sewn in 13 patients (43%). In 67% of the patients, an end-to-end anastomosis was made. In 24 patients (80%), the surgeon decided to create a primary deviating ileostomy. Intraoperative complications included a case of bleeding not requiring blood transfusion and one incomplete donut after stapling requiring a circular hand sewn anastomosis. Specimen retrieval was done through a Pfannenstiel incision (60%), trocar/stoma site (7%) or transanally (33%). There were no conversions.

### Pathology

All specimens (100%) were R0, defined as no tumor tissue within 1 mm of the resection margins. One specimen was judged incomplete (3.3%). Positive lymph nodes were found in 7 (23%) patients. The mean number of harvested lymph nodes was 15 (IQR 9–18). Stage according to the AJCC is shown in Table 1.

### Postoperative outcome

Overall 30-day morbidity was 36.7% including major morbidity (Clavien–Dindo grade ≥III) in 17% of the patients. Two patients (7%) developed anastomotic leakage requiring reoperation. One of these patients already had a diverting ileostomy. Two patients had a presacral abscess for which CT guided drainage was performed. Both patients already had a diverting ileostomy. Stoma-related problems included high output ileostomy (21%) requiring medication and passage problems (8%) requiring re-intervention. Three patients needed temporary catheterization for urinary retention. Median hospital stay was 7 days (IQR 6–10 days).

### Long-term outcome

Median follow-up duration was 14 months (IQR 8–19 months). No local recurrence was observed during follow-up. Distant metastases were found in 5 patients (17%) with a median interval of 185 days (IQR 62–239 days) after initial surgery. In 3 patients, liver metastases were treated with radical resection or radio-frequency ablation (RFA). In 1 patient, treated for local recurrence after wait and see policy for a complete response failed, palliative treatment was started when widespread pulmonary, para-aortal, and peritoneal metastases were observed. One patient developed brain metastases and died 7 months postoperatively.

### Overall quality of life (EQ-5D-3L)

The preoperative mean score of the EQ-5D index was 90.2 (95% CI 83.9–96.5). After 1 month a significant decrease was observed to 78.2 (95% CI 68.9–87.4), (*p* = 0.031), and after 6 months, the EQ-5D returned near to preoperative score, 86.0 (95% CI 79.9–92.2). Subanalysis of the domains showed a significant increase in problems experienced in social life (*p* = 0.015) at 1 month, but this significant difference disappeared after 6 months. The other domains were not significantly influenced by the operation (Table 2). Analyzing the EQ-VAS for pain, a significant aggravation was observed 1 month after surgery (*p* = 0.008), but the VAS scores were similar to the other scores after 6 months when no significant difference was found compared to baseline (*p* = 0.351).

**Table 2:** EQ-5D

	Preoperative	1 month	6 months
EQ-5D index (mean, 95% CI) *p* = 0.031^¥^	90.2 (83.9–96.5)	78.2 (68.9–87.4) *p* = 0.094^×^	86.0 (79.9–92.2) *p* = 0.894^×^
VAS (mean, 95% CI) *p* = 0.002^¥^	82.5 (78.1–86.9)	70.0 (63.4–76.5) *p* = 0.008^×^	77.5 (72.3–82.7) *p* = 0.351^×^
Mobility			
Level I	93	80	82
Level II	7	20	18
Level III	0	0	0
ADL			
Level I	100	93	100
Level II	0	7	0
Level III	0	0	0
Social life			
Level I	87	37	71
Level II	13	43	22
Level III	0	20	7
Experienced pain			
Level I	77	47	63
Level II	23	47	37
Level III	0	6	0
Mood			
Level I	77	83	78
Level II	20	17	22
Level III	3	0	0

All data in this table are presented as percentages unless explained otherwise

*VAS* visual analogue scale, *CI* confidence interval, *ADL* activities of daily life

^¥^ *p* value of repeated measures ANOVA

^×^ *p* = value of post hoc analysis performed when repeated measures ANOVA was significant

### Colorectal cancer specific quality of life (EORTC QLQ-C30 and QLQ-CR29)

**Fig. 2:**
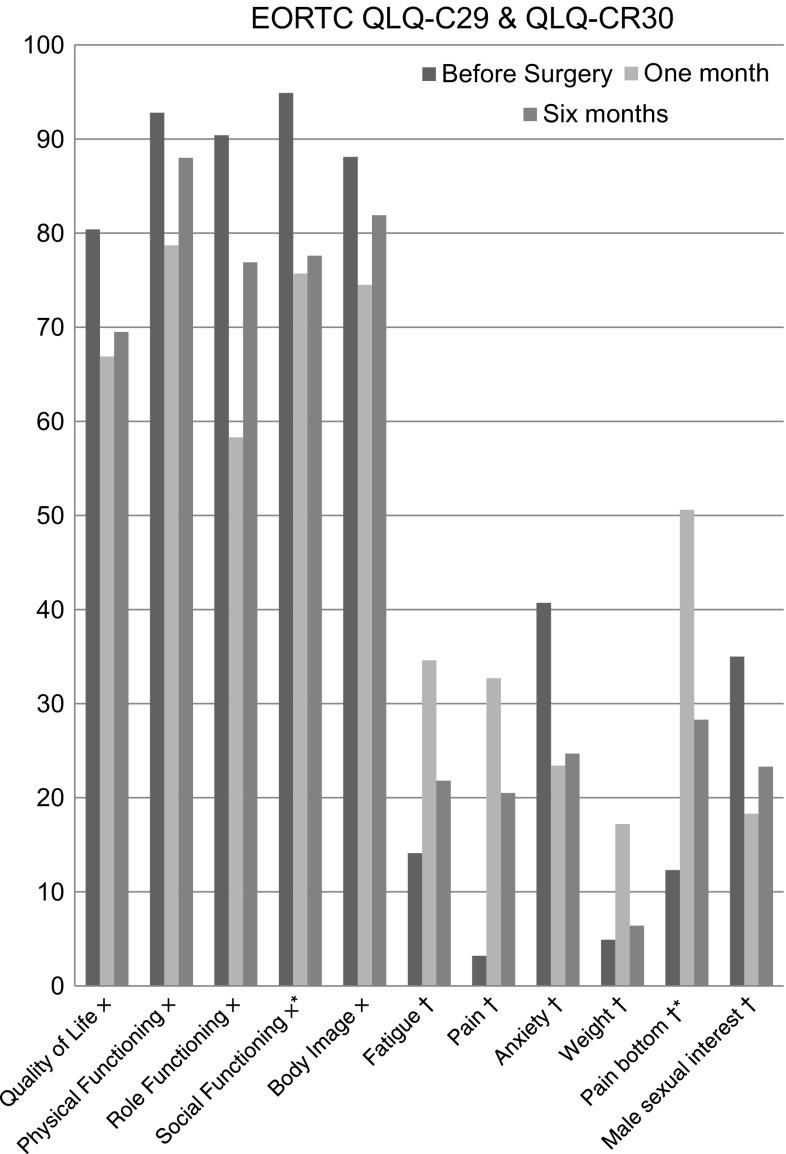
Domains measured by the EORTC QLQ-C29 and QLQ-CR30 questionnaires, at inclusion, and after 1 and 6 months. Mean scores are shown. Only domains that changed significantly are displayed. *Asterisk* significant difference at 6 months as compared to before surgery. *Multiplication sign* a high value is positive to the patient; *dagger* a high value is negative to the patient

Significant drop in scores was observed at 1 month after surgery for quality of life (*p* = 0.012), physical functioning (*p* = 0.001), role functioning (*p* < 0.001), fatigue (*p* = 0.002) and general pain (*p* = 0.001). After 6 months, the effect of TaTME disappeared for these scores, except for social functioning (*p* = 0.013) and anal pain (*p* = 0.013), which at 6 months remained significantly lower than preoperative scores (Fig. 2).

### Bladder function

Symptoms of urinary incontinence, increased frequency or dysuria did not change significantly after TaTME (*p* = 0.425, *p* = 0.618 and *p* = 0.146, respectively). Mean scores preoperatively were 3.7 (95% confidence interval (CI) −0.5 to 7.9), 28.4 (95% CI 20.7–36.1), and 0.0 (95% CI 0.0), respectively. The mean difference compared to preoperative scores remained below 5.0 points at 1 and 6 months.

### Ano(neo)rectal function after stoma closure

The mean preoperative LARS score was 15.4 (95% CI 7.3–23.5). At 1 month, this score significantly increased to 35.7 (95% CI 32.9–38.6), (*p* = 0.001). At 6 months, the mean score fell to 21.7 (95% CI 13.6–29.9). The mean difference between preoperative and 6-month values was not significant (*p* = 0.339). A total of 33% of the included patients reported symptoms correlating with major LARS at 6 months. Descriptive values of LARS are shown in Table 3.

**Table 3:** LARS

	Before	1 month	6 months
Incontinence for flatus			
Never	73.3	13.3	40.0
<Once a week	20.0	26.7	26.7
≥Once a week	6.7	60.0	33.3
Incontinence for liquid stools			
Never	80.0	26.7	40.0
<Once a week	13.3	33.3	26.7
≥ Once a week	6.7	40.0	33.3
Frequency bowel			
1–3 times a day	46.7	26.7	26.7
4–7 times a day	20.0	40.0	46.7
>7 times a day	13.3	33.3	20.0
<Once a day	20.0	0	6.7
Clustering of stools			
Never	53.3	0	26.7
<Once a week	20.0	13.3	33.3
≥Once a week	26.7	86.7	40.0
Urgency			
Never	53.3	0	46.7
<Once a week	13.3	20.0	26.7
≥Once a week	33.3	80.0	26.7
LARS (mean, 95% CI) *p* < 0.001^¥^	15.4 (7.3–23.5)	35.7 (32.9–38.6) *p* = 0.001^×^	21.7 (13.6–29.9) *p* = 0.339^×^
No LARS	53.3	0	46.7
Minor LARS	33.3	20.0	20.0
Major LARS	13.4	80.0	33.3

All data in this table are presented as percentages unless explained otherwise

*LARS* low anterior resection syndrome, *CI* confidence interval

^¥^ *p* value of repeated measures ANOVA

^×^ *p* = value of post hoc analysis performed when repeated measures ANOVA was significant

### Sexual function

Male interest in sexual intercourse significantly decreased at 1 month postoperatively, but returned to the same level as before surgery at 6 months. Erection problems did not increase significantly after surgery (completion rate 57%). Only two female patients answered the question about sexual interest, and no female patient answered the question about dyspareunia.

## Discussion

To our knowledge, this is the first study to present preoperative and postoperative reported quality of life by patients undergoing TaTME low anterior resection for rectal cancer using prospectively gathered validated questionnaires. One month after TaTME significantly decreased, scores were reported for EQ-5D, EQ-VAS, quality of life, physical functioning, role functioning, social functioning, fatigue, general experienced pain, anal pain, LARS, and male sexual interest. After 6 months, most scores returned to baseline values except for social functioning and anal pain, though mean values did improve. Major LARS was present in 33% of the patients 6 months after stoma reversal.

The study by Andersson et al. [19, 20] on health-related quality of life using data from the COLOR II trial, one of the largest randomized trials evaluating laparoscopic and open rectal cancer resection, showed meaningful clinical changes in quality of life at 1 month after conventional rectal cancer surgery, but values returned to (or close to) preoperative values by 6 months after surgery. These results are comparable to the quality of life scores found in our study using the same questionnaires (EQ-5D-3L and EORTC QLQ-C30) and study design.

Major LARS is associated with an immense decrease in quality of life [20] and is considered one of the most important functional outcome measurements after rectal surgery. Kneist et al. [12] were the first authors who reported LARS after TaTME for low rectal cancer in 10 patients and showed that 40% of the patients experienced no LARS, 50% minor LARS and only 10% major LARS 6 months after stoma closure. However, Pontallier et al. [21] reported major LARS 12 months after stoma closure in 82% of the patients with coloanal anastomosis. In our study, the outcome of 15 patients showed 47% no LARS, 20% minor LARS and 33% major LARS. This percentage for major LARS after TaTME in this study is lower than the published scores found after conventional TME, which are often reported around 50% [22–24]. This cohort included 1 patient after anastomotic leakage who had an adequate period of follow-up after reversal of the stoma. Function of the other 3 patients with an anastomotic leakage or presacral abscess is to be awaited and potentially could negatively influence the LARS score found in our cohort [25, 26]. On the other hand, our cohort analyzing anorectal function contains a high amount of patients with an end-to-end anastomosis (73%), which is a known risk factor for worse functional outcome on the short term after rectal resection.

In our study, 3 patients (10%) developed urinary retention postoperatively for which they needed temporary catheterization. Patient-reported outcome on micturition impairment at 4 weeks showed no significant increase, which might imply good bladder function due to sparing of the nerves. Our results after TaTME, describing urinary retention requiring temporary catheterization in up to 10% of patients, are comparable to those in the literature [8–12]. Permanent micturition problems, like incontinence, retention or increased voiding frequency have not been reported after TaTME, as confirmed by the results of our study.

Decrease of sexual interest was observed at 1 month but at 6 months this almost returned to preoperative values. No significant erectile malfunction was seen compared to baseline. Tuech and Kneist [11, 12] examined male sexual function after TaTME and reported 11–22% impotence, 22% worse erectile function and 33% decreased ejaculation. Pontallier et al. described laparoscopic surgery as an independent risk factor for loss of sexual activity. No significant difference was found in sexual function between TaTME and laparoscopic TME [21].

Results on female patients after TaTME remain too scarce to permit any solid conclusions. We included only eight females and due to the low completion rate of the questionnaires by these patients, valid statistical analysis was not possible.

Four patients who underwent TaTME through APR were excluded in order to minimize the heterogeneity of the study group. In the literature, the effects of a permanent stoma on quality of life are a topic of debate. How et al. and Kasparek et al. [27, 28] reported that this effect was not significant and quality of life was similar between patients who underwent low anterior resection and APR. Quality of life after APR with distal extralevator or intersphincteric transanal resection is still to be evaluated.

Our study results are obviously limited due to the small sample size, great heterogeneity of the study group and wide confidence intervals. Another limitation in this single-center study is that no adequate comparison group could be presented since all patients undergoing elective surgery for mid- or low rectal cancer during the inclusion period were operated on using TaTME. Therefore, this single-center study provides exploratory findings rather than conclusive evidence. Moreover, our follow-up was limited to 6 months since earlier studies evaluating health-related quality of life after conventional surgery showed that significant changes disappeared at 6 months after surgery [18, 19]. The Wexner score, IPSS and IIEF-5 were not used since most questions were already included in the questionnaires employed and in order to prevent a lower response rate due to a larger number of questions asked at the different time points. Only univariate analysis was performed due to the small sample size; therefore, we were not able to discriminate any potential risk factors for decreased quality of life or impaired function including type of anastomosis, anastomotic leakage, neoadjuvant therapy or level of the anastomosis, factors which have been observed to influence functional outcome after conventional rectal resection [20, 25, 26, 29].

Despite these limitations, this study provides the first data on quality of life and functional outcome at 1 and 6 months after TaTME compared to preoperative values showing results comparable to published results after laparoscopic abdominal TME. Extra focus on postoperative pain management and training of pelvic floor muscles might be suggested to improve social functioning within 6 months after TaTME. The COLOR III trial, a randomized clinical trial comparing laparoscopic TME with TaTME, will show robust data exploring quality of life, functional results and all relevant parameters influencing patient-reported outcome after mid- and low restorative rectal cancer surgery [30].

## Conclusions

Using validated questionnaires, TaTME does not appear to substantially impair functional and quality of life outcomes compared to laparoscopic abdominal TME. Further studies are needed to confirm these results.

## References

[1] Lacy AM, Adelsdorfer C, Delgado S, Sylla P, Rattner DW (2013). Minilaparoscopy-assisted transrectal low anterior resection (LAR): a preliminary study. Surg Endosc.

[2] Velthuis S, van den Boezem PB, van der Peet DL, Cuesta MA, Sietses C (2013). Feasibility study of transanal mesorectal excision. Br J Surg.

[3] Lacy AM, Tasende MM, Delgado S (2015). Transanal total mesorectal excision for rectal cancer: outcomes after 140 patients. J Am Coll Surg.

[4] Veltcamp Helbach M, Deijen CL, Velthuis S, Bonjer HJ, Tuynman JB, Sietses C (2015). Transanal total mesorectal excision for rectal carcinoma: short-term outcomes and experience after 80 cases. Surg Endosc.

[5] Simillis C, Hompes R, Penna M, Rasheed S, Tekkis PP (2015). A systematic review of transanal total mesorectal excision: is this the future of rectal cancer surgery?. Colorectal Dis.

[6] Deijen CL, Tsai A, Koedam TW (2016). Clinical outcomes and case volume effect of transanal total mesorectal excision for rectal cancer: a systematic review. Tech Coloproctol.

[7] Bonjer HJ, Deijen CL, Abis GA (2015). A randomized trial of laparoscopic versus open surgery for rectal cancer. N Engl J Med.

[8] Rouanet P, Mourregot A, Azar CC (2013). Transanal endoscopic proctectomy: an innovative procedure for difficult resection of rectal tumors in men with narrow pelvis. Dis Colon Rectum.

[9] Atallah S, Martin-Perez B, Albert M (2014). Transanal minimally invasive surgery for total mesorectal excision (TAMIS-TME): results and experience with the first 20 patients undergoing curative-intent rectal cancer surgery at a single institution. Tech Coloproctol.

[10] Elmore U, Fumagalli Romario U, Vignali A, Sosa MF, Angiolini MR, Rosati R (2015). Laparoscopic anterior resection with transanal total mesorectal excision for rectal cancer: preliminary experience and impact on postoperative bowel function. J Laparopendosc Adv Surg Tech A.

[11] Tuech J, Karoui M, Lelong B (2015). A step towards NOTES total mesorectal excision for rectal cancer. Ann Surg.

[12] Kneist W, Wachter N, Paschold M, Kauff D, Rink A, Lang H (2016). Midterm functional results of taTME with neuromapping for low rectal cancer. Tech Coloproctol.

[13] Rahbari N, Weitz J, Hohenberger W (2010). Definition and grading of anastomotic leakage following anterior resection of the rectum: a proposal by the International Study Group of Rectal Cancer. Surgery.

[14] Quirke P, Steele R, Monson J, MRC CR07/NCIC-CTG CO16 Trial Investigators; NCRI Colorectal Cancer Study Group (2009). Effect of the plane of surgery achieved on local recurrence in patients with operable rectal cancer: a prospective study using data from the MRC CR07 and NCIC-CTG CO16 randomised clinical trial. Lancet.

[15] Whistance RN, Conroy T, Chie W (2009). Clinical and psychometric validation of the EORTC QLQ-CR29 questionnaire module to assess health related quality of life in patients with colorectal cancer. Eur J Cancer.

[16] EORTC Quality of Life Group. The EORTC QLQ-C30 Scoring Manual (3rd Edition) 2001

[17] Emmertsen KJ, Laurberg S; Rectal Cancer Function Study Group (2012). Low anterior resection syndrome score: development and validation of a symptom-based scoring system for bowel dysfunction after low anterior resection for rectal cancer. Ann Surg.

[18] Osoba D, Rodrigues G, Myles J, Zee B, Pater J (1998). Interpreting the significance of changes in health-related quality-of-life scores. J Clin Oncol.

[19] Andersson J, Angenete E, Gellerstedt M (2013). Health-related quality of life after laparoscopic and open surgery for rectal cancer in a randomized trial. Br J Surg.

[20] Andersson J, Abis G, Gellerstedt M (2014). Patient-reported genitourinary dysfunction after laparoscopic and open rectal cancer surgery in a randomized trial (COLOR II). Br J Surg.

[21] Pontallier A, Denost Q, Van Geluwe B, Adam JP, Celerier B, Rullier E (2016). Potential sexual function improvement by using transanal mesorectal approach for laparoscopic low rectal cancer excision. Surg Endosc.

[22] Emmertsen KJ, Laurberg S; Rectal Cancer Function Study Group (2013). Impact of bowel dysfunction on quality of life after sphincter-preserving resection for rectal cancer. Br J Surg.

[23] Ekkarat P, Boonpipattanapon T, Tantiphlachiva K (2015). Factors determining low anterior resection syndrome after rectal cancer resection: a study in Thai patients. Asian J Surg.

[24] Juul T, Ahlberg M, Biondo S (2014). Low anterior resection syndrome and quality of life: an international multicenter study. Dis Colon Rectum.

[25] Bregendahl S, Emmertsen KJ, Lous J, Laurberg S (2013). Bowel dysfunction after low anterior resection with and without neoadjuvant therapy for rectal cancer: a population-based cross-sectional study. Colorectal Dis.

[26] Nesbakken A, Nygaard K, Lunde OC (2001). Outcome and late functional results after anastomotic leakage following mesorectal excision for rectal cancer. Br J Surg.

[27] How P, Stelzner S, Branagan G (2012). Comparative quality of life in patients following abdominoperineal excision and low anterior resection for low rectal cancer. Dis Colon Rectum.

[28] Kasparek MS, Hassan I, Cima RR, Larson DR, Gullerud RE, Wolff BG (2012). Long-term quality of life and sexual and urinary function after abdominoperineal resection for distal rectal cancer. Dis Colon Rectum.

[29] Guren MG, Eriksen MT, Wiig JN (2005). Quality of life and functional outcome following anterior or abdominoperineal resection for rectal cancer. Eur J Surg Oncol.

[30] Deijen CL, Velthuis S, Tsai A (2016). COLOR III: a multicenter randomised clinical trial comparing transanal TME versus laparoscopic TME for mid and low rectal cancer. Surg Endosc.

